# Towards Attention-Based Convolutional Long Short-Term Memory for Travel Time Prediction of Bus Journeys

**DOI:** 10.3390/s20123354

**Published:** 2020-06-12

**Authors:** Jianqing Wu, Qiang Wu, Jun Shen, Chen Cai

**Affiliations:** 1 School of Computing and Information Technology, University of Wollongong, Wollongong, NSW 2522, Australia; jw937@uowmail.edu.au; 2 School of Information and Engineering, Lanzhou University, Lanzhou 730000, China; wuq17@lzu.edu.cn; 3 Data 61, CSIRO, Eveleigh, NSW 2015, Australia; chen.cai@data61.csiro.au

**Keywords:** travel time prediction, bus journey, convolutional long short-term memory, attention mechanism

## Abstract

Travel time prediction is critical for advanced traveler information systems (ATISs), which provides valuable information for enhancing the efficiency and effectiveness of the urban transportation systems. However, in the area of bus trips, existing studies have focused on directly using the structured data to predict travel time for a single bus trip. For state-of-the-art public transportation information systems, a bus journey generally has multiple bus trips. Additionally, due to the lack of study on data fusion, it is even inadequate for the development of underlying intelligent transportation systems. In this paper, we propose a novel framework for a hybrid data-driven travel time prediction model for bus journeys based on open data. We explore a convolutional long short-term memory (ConvLSTM) model with a self-attention mechanism that accurately predicts the running time of each segment of the trips and the waiting time at each station. The model is more robust to capture long-range dependence in time series data as well.

## 1. Introduction

The usage of intelligent transportation systems (ITSs) is motivated in a significant part by passenger increases and sustainable development [1,2]. The ITS has a direct impact on energy consumption, personal living expenses, public health and safety. Seamless integration of vehicles and sensing devices has made it possible to capture and collect large amounts of sensor data from various data sources in real time. Developing sustainable and intelligent transportation applications operate and manage real-time and historical data efficiently, which has become an increasingly important yet challenging task. It also plays a vital role in achieving the main objectives of ITS, which include accessibility and mobility, environmental sustainability and economic development [3,4]. With the advent of artificial intelligence (AI), machine learning and expert system-based paradigms have driven the development of society and the steady growth of the economy. Besides, deep learning can discover patterns in complex data sets, which could not be found via conventional methods. The merging of machine learning and transportation science has tremendous potential to enhance the performance of ITS.

Travel time refers to a period spent traveling from the origin to the destination. Providing real-time travel information is indispensable for ITS. However, real-time travel time is unlikely to be observed because it is already historical data rather than ‘real-time data’ since it was collected [5]. Using predictive methods to estimate future travel time is an effective way to provide real-time information. Furthermore, travel time prediction is a known and challenging research area because of the inherent uncertainty [6]. Existing studies on bus travel time prediction mainly focus on improving the prediction accuracy of a single trip. This is inadequate for implementing efficient applications in an intelligent transportation system, where a bus journey has multiple bus trips [7]. Although the ConvLSTM has shown excellent performance in travel time prediction, adding the attention mechanism to LSTM-based models has the potential to improve the predictive accuracy [8,9]. The integration of their strengths remains an unsolved research task. Studies have applied LSTM-based deep learning methods with applications to journey travel time prediction that rely on high-quality labeled data. However, data acquisition is a challenging task.

The contributions of this study are summarized as follows:

(1) We designed and developed an open-source data collection framework that can automatically collect and pre-process large amounts of high-quality data over a long period without involving personal privacy, for example, an entire season or even several years.
(2) This paper proposes a hybrid model that applies the ConvLSTM network with an attention mechanism to explore a suitable model for the bus journey time prediction on open data.
(3) We also discuss input features for journey travel time prediction and suggest directions for future research.

The remainder of the paper is organized as follows. Firstly, we demonstrate a brief overview of the basic definitions. Secondly, an integrated system framework is introduced to target the problem of bus journey time prediction and provides a ConvLSTM-based method with self-attention. Furthermore, the datasets’ baseline and evaluation metrics are used in this study. Finally, the findings and suggestions for further studies are summarized.

## 2. Related Works

The sustainable development of *smart cities* requires reliable and efficient transportation systems [10]. Internet of Things (IoT) can be applied with the existing infrastructure and service networks for the design of transportation systems, such as software-defined networks and communication technologies [11,12,13]. IoT-based intelligent transportation systems (IoT-ITSs) can be classified into four main fields: Advanced traveler information system (ATIS), advanced public transportation system (APTS), advanced traffic management system (ATMS) and emergency management system (EMS) [13]. Transportation systems are shifting from conventional technology-driven systems to more powerful multifunctional data-driven ITSs [14,15,16]. Massive traffic sensor data gathered by various sensors are vital for informed scientific decision-making processes in traffic operation, pavement design and transportation planning [17]. Data analytics in ITSs consider important factors that influence decision-making processes, such as travel time or traffic congestion of public transport services [18,19]. The fusion of traffic data from multiple sources produces a better understanding of the observations to reach a better inference in ITSs [20,21,22,23].

Accurate estimation of travel time is essential to the success of ATMS and ATIS [24]. The approaches to studying travel time prediction can be mainly divided into three categories: Knowledge-driven, model-driven and data-driven approaches. Knowledge-driven approaches usually employ a database, a knowledge base in the form of rules and an inference engine in the form of algorithms [25]. Lee et al. proposed a knowledge-based expert system that predicted travel time by combining general rules from location-based service applications and meta-rules from human domain experts [26]. Nonetheless, as the knowledge base becomes increasingly large, the time to obtain accurate predictions increases as well. Model-driven approaches can be divided into four levels: Macroscopic (e.g., TOPL [27]), mesoscopic (e.g., DynaMIT [28] and Dynasmart [29]), cellular automaton (CA) (e.g., OLSIM [30]) and microscopic methods (e.g., AIMSUM online [31]) [32]. In the past, most of the studies on travel time forecasting have focused on model-based methods. Transport simulation software is intended for simulating traffic state information on virtual networks. It is primarily focused on research in traffic control and management, such as the effects of ramp metering, variable speed limits and traffic incidents. To perform research on model-based practices, we need to acquire and use travel demand data, which is known as an origin-destination (OD) matrix or population data [5]. Nevertheless, accurate OD data is difficult to obtain, time-consuming and expensive. Presently, only a few institutions have accumulated essentially useful OD data to build integrated travel time forecasting systems.

Recently, data-driven approaches have been receiving increased attention and gained interest within the transportation research community due to the increased computing power available and the vast amount of data collected in ITSs. Deep learning leads to an advantage over conventional machine learning algorithms with big data analytics of urban traffic. Kumar et al. compared the performance of the data-driven artificial neural network (ANN) approach and the model-based Kalman filter (KF) approach concerning bus travel time prediction in [33]. The experimental results showed that the data-driven ANN can achieve better performance, but compared to KF, the model needs a rich set of data for neural network training. Hou and Edara proposed long short-term memory (LSTM) and convolutional neural network (CNN) to predict travel time in a road network; compared to CNN, random forests (RFs) and gradient boosting machines (GBMs); the computation time of LSTM was the shortest in the model training process and prediction process [34]. Petersen et al. utilized the convolutional LSTM to propose a multi-output multi-time-step system for bus travel time prediction [8]. Yu et al. presented a random forest based on the near neighbor (RFNN) model to predict the travel times of buses between bus stops, which include the running time and waiting time as two input variables separately. Correspondingly, the model also considers traffic conditions, which is an essential factor affecting bus travel time [35]. However, studies on bus journey time forecasting is rather limited. Our work focuses on forecasting the travel time of the bus journey for travelers. A trip is to use one transport mode to travel on a single line or route, and a journey has one or more trips, where transfers occur between bus services during a period of travel time [7]. Therefore, there is still a need for developing a well-designed system framework to discover the advantages of various methods that achieve a deterministic and meaningful outcome, which is closer to the real world’s needs.

However, none of the existing studies have considered the travel time problem of a bus journey via the ConvLSTM with the self-attention mechanism. Thus, the objective of our study was to predict the travel time of bus journeys by leveraging a data fusion component, which offers appropriate inputs to deep learning models.

## 3. Methodology

### 3.1. Bus Travel Time

In this section, we define some terms in Table 1, which will be used throughout the rest of the paper.

**Figure 1:**
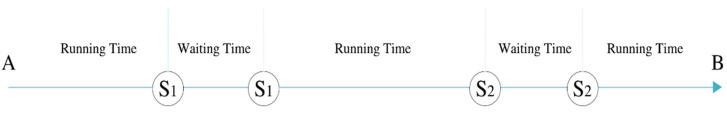
Running time and waiting time for a bus trip.

A bus usually runs along a fixed route based on a regular schedule. The travel time depicted in Figure 1 is the time cost to complete a trip, which departs at time *t*. It follows an itinerary characterized by an original station A, a destination station B and some stops (e.g., station $S_{1}$ and station $S_{2}$).

In this paper, we predict the total travel time of a bus journey by using the actual running time and waiting time from open data. For any stops in the trip, a bus is scheduled to arrive and depart from a stop *S* at different specified times, defined in the timetable, respectively, $t_{d}T,S$ and $t_{a}T,S$. In general, travel time forecasting is an estimate of the trip from a station of origin to a station of destination. The running time is the absolute difference between the arrival time of the current station and the departure time of the previous station, such as $R_{2}=t_{a}T,S_{2}-t_{d}T,S_{1}$. The waiting time is the absolute difference between the departure time and the arrival time in a fixed stop station, such as, $D_{1}=t_{d}T,S_{1}-t_{a}T,S_{1}.$ Our study defines segments based on information about the stops of a trip pattern. The segment-based method divides the stop points into running time and waiting time segments. Our predictive models predict the running and waiting times based on different $t_{a}$ and $t_{d}$. According to Figure 1, it is evident that the numbers of input data for the prediction of running time and waiting time are different. This is because for each trip of a specific bus, the running time will have one more record than the waiting time. The total travel time of a bus journey can be described with Equation (1):

(1) $$t_{total}=\sum_{i}^{n}\hat{R}+\sum_{i}^{n-1}{\hat{D}}_{i}.$$

### 3.2. Leveraging Machine Learning and Logical Reasoning

With the rapid development of ITSs in recent years, data availability issues have always plagued researchers. Notably, the studies of multi-modal transport require a large amount of data from diverse data sources. Open data platforms release a variety of data that is freely available to everyone to reuse. Moreover, domain experts structure and classify data, such as general transit feed specification (GTFS) and GTFS-Realtime [36]. Researchers can create structured data, namely the process of data curation, for the corresponding studies through data cleansing and data fusion. To predict a complex and uncertain event, we need to have multiple sources of data to provide more information for generating a predictive model.

**Figure 2:**
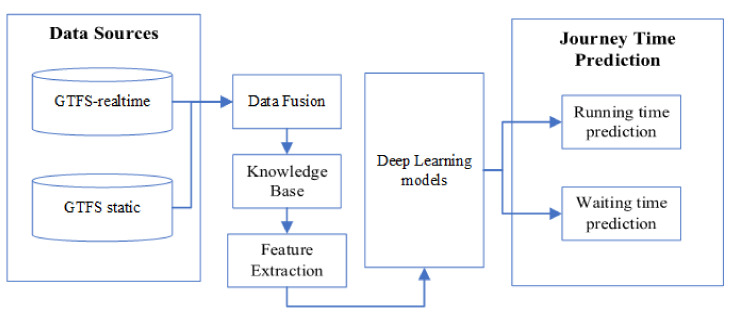
The framework of journey time prediction.

Figure 2 illustrates the framework of an integrated system for journey time prediction, which consists of six components: GTFS-Realtime and GTFS static data stores, data fusion, knowledge base, feature extraction, deep learning models, and running time prediction and waiting time prediction. As Figure 2 shows, in the first step, we collected data from two types of GTFS and cleansed them, for example, by deleting duplicate data and sorting the data in chronological order. In order to build a knowledge base, the data fusion approach plays an essential role. Data from different data sources sometimes cannot be integrated and saved into a relational database or a two-dimensional data format, due to some data failing to match one-to-one or one-to-many mapping relationships, such as the running time from the station $S_{1}$ to $S_{2}$ and probe vehicle speed data. The use of the knowledge base enables deep learning models to exploit logical reasoning from data. Applying domain knowledge to classify the raw data not only avoids the impact of irrelevant data but also reduces the computation time of the model. Furthermore, data fusion employs mathematical methods and programming languages to synthesize useful information or inferences. The theoretical framework can also be developed as an extended version to involve verification mechanisms [37].

### 3.3. Bus Journey Travel Time with Multi-Step Time Series Prediction

The ConvLSTM model is a powerful kind of recurrent neural network (RNN), with a combination of convolutional and LSTM layers, which contains the operation inside the LSTM cell [38]. On the other hand, the travel time prediction of a bus journey can be treated as a time series prediction problem. In recent years, LSTM is an elegant solution to the time series analysis by exploiting spatiotemporal data. Additionally, the ConvLSTM applies the convolution operators to capture the spatial and temporal dependencies in the dataset so that it generally performs better than fully connected LSTM (FC-LSTM) [38]. The calculation steps are as follows:

Firstly, calculate the input gate:

(2) $$i_{t}=\sigma \left(W_{xi}\times \;x_{t}+W_{hi}\times h_{t-1}+W_{ci}\circ c_{t-1}+b_{i}\right),$$

Forget gate:

(3) $$f_{t}=\sigma \left(W_{xf}\;\times x_{t}+W_{hf}\times h_{t-1}+W_{cf}\circ c_{t-1}+b_{f}\right),$$

Cell state:

(4) $$c_{t}=f_{t}\circ c_{t-1}+i_{t}\circ tanhW_{xc}\times x_{t}+W_{hc}\times h_{t-1}+b_{c},$$

Output gate:

(5) $$o_{t}=\sigma \left(W_{xo}\times \;x_{t}+W_{ho}\times h_{t-1}+W_{co}\circ c_{t}+b_{o}\right),$$

Hidden state:

(6) $$h_{t}=o_{t}\circ tanh\left(c_{t}\right),$$

where σ is a sigmoid function, ∘ is the Hadamard product, and × is the convolution operator. $W_{xi}$, $W_{xf}$, $W_{xc}$ and $W_{xo}$ are the weight matrices connecting the inputs $x_{1}$, …, $x_{t}$ to three gates and the cell input; $W_{hi}$, $W_{hf}$, $W_{hc}$ and $W_{ho}$ are the weight matrices connecting the hidden states $h_{1}$, …, $h_{t-1}$ to three gates and the cell input; $W_{ci}$, $W_{cf}$ and $W_{co}$ are the weight matrices connecting the $c_{1}$, …, $c_{t}$ to three gates; and $b_{i}$, $b_{f}$, $b_{c}$ and $b_{o}$ are the bias terms of three gates and the cell state.

Recently, the attention mechanism has succeeded in a wide range of sequence-to-sequence learning tasks [39,40,41]. Liang et al. presented a multi-level attention-based recurrent neural network for predicting geo-sensory time series [42]. The attention model focuses on the vital issue with the LSTM-based model for bus travel time prediction, which tends to select near-term data that is highly correlated to future travel time. In our experiments, the encoder is the underlying ConvLSTM model generating the hidden state representation $h_{t}$. We leverage a self-attention mechanism to the inputs after the operations of Equations (1)–(6):

(7) $$m_{t,t^{\prime }}=tanh\left(W_{m}h_{t}+W_{m^{\prime }}h_{t^{\prime }}+b_{m}\right),$$

(8) $$e_{t,t^{\prime }}=\sigma \left(W_{a}m_{t,t^{\prime }}+b_{a}\right),$$

(9) $$a_{t}=softmax\left(e_{t}\right),$$

(10) $$l_{t}=\overset{n}{\underset{t^{\prime }=1}{\sum }}a_{t,t^{\prime }}\times h_{t^{\prime }},$$

where $a_{t,t^{\prime }}$ is an attention matrix; $b_{m}$ and $b_{a}$ express bias terms; $W_{m}$ and $W_{m^{\prime }}$ express weight matrices corresponding to the hidden states $h_{t}$, $h_{t^{\prime }}$; and finally, $l_{t}$ represents a weighted sum of $h_{t^{\prime }}$ [43].

**Figure 3:**
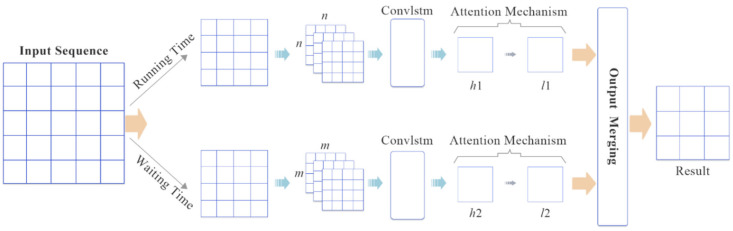
Self-attention-based ConvLSTM network.

Figure 3 demonstrates an overview of our proposed model, which consists of two main components: Running time prediction and waiting time prediction, which are two independent components for estimating running and waiting times based on GTFS-Realtime. The first step is to divide the historical observations from a sequence dataset into two smaller sequence datasets so that the input data of the ConvLSTM model are arranged into a 3-D-tensor for a single bus line. For example, in *N* day samples and time steps k, a sequence of running times $R_{i}$ with a single bus line can be represented as (*N*, k, $R_{i}$). Secondly, $l_{1}\;and\;l_{2}\;$ show how much the weight of the historical observations affects the predicted values. Finally, the outputs are merged to get the results by using Equation (1).

The entire training process of an attention ConvLSTM is presented in Algorithm 1. We firstly construct multiple historical observation sequences as inputs. Then, the model is trained to predict the running time and waiting time separately.

**Algorithm 1** Attention-Based ConvLSTM Training AlgorithmRequire:Historical running time and waiting time observations:
$\left(R_{1}^{T},R_{2}^{T}\ldots R_{n}^{T}\right)\;and\;\left(D_{1}^{T},D_{2}^{T}\ldots D_{n-1}^{T}\right);$
Sequence lenght: *n*;Lengths of running time, waiting time: *l_R_*, *l_D_*;running time: *R*;waiting time: *D*.Ensure: Attention-based ConvLSTM Modelfor *epoch* = *max–epoch* doPerform forward propagation recurrently using Equation (2)–(10) tocalculate
$S_{R}=\left(R_{1}^{T},R_{2}^{T}∆R_{n}^{T}\right)$

$S_{D}=\left(D_{1}^{T},D_{2}^{T}∆D_{n}^{T}\right)$
compute output error:
$Y_{R\;}-{\hat{Y}}_{R}$

$Y_{D\;}-{\hat{Y}}_{D}$
merging the predicted outputs to obtain the total travel time:
$t_{total}={\hat{Y}}_{R}+{\hat{Y}}_{D}$
end for

## 4. Experiments and Discussion

### 4.1. Dataset Description and Preprocessing

We verified our model on real-world traffic datasets from TfNSW (Transport for NSW) Open Data Bus Realtime Trip Update (BRTU) collected by a Python program that read the TfNSW real-time feed application programming interfaces (APIs) [44]. The dataset contains key attributes of bus journey information with corresponding timestamps, as detailed below.

BRTU was gathered from Sydney’s bus system in real time. For our experiment, the data was collected every 60 s, about 12 GB of data a day. Note that the better frequency is 10 s, around 60 GB a day). The period used was from 6th May 2019 to 28th June 2019 except the weekends. We selected the first three weeks of historical travel time records as a training set and the rest served as a test set, respectively. BRTU has information about the departure time, arrival time, delay and route. The GTFS-static contains station names, coordinates and route names.

The proposed model and other comparative models were implemented in Python via the TensorFlow Framework [45] and trained with the Adam algorithm [46]. The proposed network was composed of several layers: A ConvLSTM2D [38], a flatten layer, a RepeatVector layer, a self-attention layer and two TimeDistributed layers. The training details about the network are presented in Table 2.

### 4.2. Evaluation Metrics and Results

In our experiments, we applied two standard metrics to evaluate the performance of running time prediction and waiting time prediction, including root mean square errors (RMSEs) and mean absolute errors (MAEs). They were defined as presented in Equations (11) and (12), where $y_{t}$ represents the actual value for sample *t* and ${\hat{y}}_{t}$ represents the predicted value. As the multi-time-step model predicts bus travel time for all stops for the next *n* time-steps, bot $y_{t}$ and ${\hat{y}}_{t}$ have the dimensionality (*N*, k, $R_{i})$:

(11) $$RMSE=\sqrt{\frac{1}{n}\sum_{i=1}^{n}{\left(y_{t}-{\hat{y}}_{t}\right)}^{2}},$$

(12) $$MAE=\frac{1}{n}\sum_{i=1}^{n}\left|y_{t}-{\hat{y}}_{t}\right|.$$

We explored the patterns of the bus running time and waiting time on weekdays. Respectively, Table 3 and Table 4 present the results of the trip id “27134” from Campbelltown station to Narellan Town Centre station. The trip “27134” has 37 records per day. As evidenced by the results, the performance of three types of LSTM does not have many differences. The output of our experiments is consistent with Greff et al.’s findings as well [47]. Standard LSTM and variant versions do not have significant performance differences.

Our design explores the pattern of each record (a stop). As can be seen from Table 3 and Table 4, we found that the attention ConvLSTM is a more stable model by observing each prediction result. It adjusts the predictions reasonably based on previous inputs. However, it cannot model very long-range temporal dependencies (e.g., period and trend), and training becomes more complicated when the depth increases [48].

Simply put, when the amount of input data increases, the time calculated by the model will increase dramatically. The attention mechanism can effectively overcome the drawbacks of modeling long-range temporal dependencies. Additionally, it could reduce the computation time in every training by using less training data.

To further verify the performance, we used LSTM and attention-based ConvLSTM to predict the running time and waiting time of one of the stops, “Mt Annan Leisure Centre, Welling Dr” (stop 18). In Table 3, a significant difference is shown. By observing each predicted value of the CNN model, we find that there is a significant difference between the upper and lower bounds for the CNN model. In this case, the prediction of the model is very unreliable. Compared with the results of LSTM models, it can be seen that the forecast results are improved in Table 3 and Table 4. Attention-based ConvLSTM’s mean errors and standard deviation (SD) are the lowest. In conclusion, attention-based ConvLSTM achieves the best overall performance compared to the other models in Table 3 and Table 4. It is a more reliable model for the prediction of travel time on data with large residuals than other models.

It is worth mentioning that our aim was not to solely improve the accuracy of predictions, as deep neural networks are less interpretable. Instead, we strived to find a practical data-driven model on open data by exploring the combination of deep learning methods and domain knowledge. Moreover, GTFS provides uncertainty values, which can be utilized to test the robustness of the generic model. The model based on GTFS will have a level of portability and reproducibility to the application in real scenarios.

**Figure 4:**
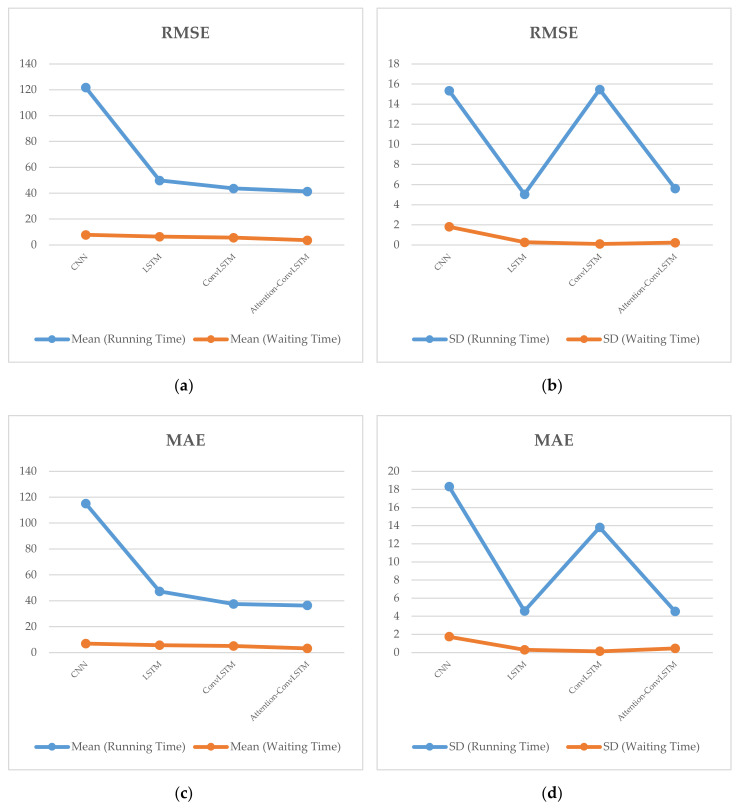
RMSE and MAE for the journey travel time prediction listed as: (**a**) The mean RMSE for the running time and waiting time; (**b**) The standard deviation of RMSE for the running time and waiting time; (**c**) The mean of MAE for running time and waiting time; (**d**) The standard deviation of MAE for the running time and waiting time.

Figure 4 reports the performance of CNN, LSTM, ConvLSTM and Attention-ConvLSTM for the prediction of the running time and waiting time. The *y*-axes of RMSE and MAE from (a), (b), (c) and (d) represent the errors in seconds, respectively. All models have significant prediction errors (mean and standard deviation) in running time predictions. Especially, CNN reaches the most significant prediction errors in all cases. The waiting times indicate small variations, which are to a great extent explained by the input in the corresponding models. A weak dependence on the journey travel time prediction is established. However, the variability of the running times cannot be fully explained by the selected input variables. Additionally, it shows that Attention-ConvLSTM effectively reduces errors. The proposed model needs to use more relevant factors to improve the predictions, such as vehicle speed or weather information.

## 5. Conclusions and Future Work

In this paper, we investigated the problem of predicting bus journeys’ travel time with publicly available GTFS data by taking into account the bus running time along routes and the waiting time at stop points. The basic idea was to use domain knowledge to classify raw data to obtain a knowledge base, which can offer useful information for assisting in deep learning models to explore the hidden patterns of data. Thus, we proposed a comprehensive framework using open data to bridge deep learning models and logical reasoning from a knowledge base. We used an attention-based ConvLSTM to predict the running time and waiting time separately. Ultimately, the total travel time prediction was obtained by merging the predicted outputs.

In the future, we will consider adding weather information, vehicle speed and traffic condition data into our deep learning models. Furthermore, we will explore evolutionary algorithms to find the best dataset size for the accurate prediction of travel time, and to find the best model number of layers and number of units per layer. According to our experiments, the use of GTFS data exchanged API will make it easier to obtain high-quality input data for multi-modal traffic prediction studies. Our future work will also focus on employing more advanced data-driven models to shift from single-mode prediction to multi-modal prediction.

## Figures and Tables

**Table 1:** List of important notations.

Symbol	Description
*T*	bus trip id *T*
*n*	number of bus stops in *T*
*S*	a bus stop in a trip *T*
$t_{d}\;$	bus departure time from the station *S*
$t_{a}\;$	bus arrival time at the station *S*
$t_{total}\;$	total time of a trip *T*
*R*	actual running time in *T*
*D*	actual waiting time in *T*
$\hat{R}$	predicted running time in *T*
$\hat{D}$	predicted waiting time in *T*
Y	actual value of evaluation metrics
$\hat{Y}$	predicted value of evaluation metrics

**Table 2:** Training details about self-attention-based ConvLSTM.

Variable	Value
learning rate	0.001
epochs	20
batch size	16
loss	Mean Squared Error
optimizer	Adam

**Table 3:** Performance comparison of the bus running time prediction models for a stop.

Models	RMSE (s)		MAE (s)	
	Mean	SD	Mean	SD
CNN	121.770	15.350	115.095	18.318
LSTM	49.849	5.046	47.146	4.583
ConvLSTM	43.720	15.468	37.533	13.821
Attention-ConvLSTM	**41.449**	5.623	**36.328**	4.539

**Table 4:** Performance comparison of the bus waiting time prediction models for a stop.

Models	RMSE (s)		MAE (s)	
	Mean	SD	Mean	SD
CNN	7.891	6.415	6.912	1.747
LSTM	6.415	0.283	5.544	0.284
ConvLSTM	5.683	0.113	5.060	0.134
Attention-ConvLSTM	**3.740**	0.227	**3.166**	0.441
